# Supplementary material for: Clinical utility of mono-exponential model diffusion weighted imaging using two b-values compared to the bi- or stretched exponential model for the diagnosis of biliary atresia in infant liver MRI
Source: PLoS One. 2019 Dec 18;14(12):e0226627. doi: 10.1371/journal.pone.0226627 (PMC6920030; doi:10.1371/journal.pone.0226627)
Supplement: S1 File — (PDF) [file pone.0226627.s001.pdf]

numb	age (v	gende	group	exam data	measure1	area (mm2	ADC	monoexpc	f	D*	D	DDC	alpha
8	12	0	0	2017-11-06		1421.875	1381.856	1379.535	143.6813	31.93368	1136.218	1346.738	729.231
9	8	0	0	2017-11-27		1486.016	1210.806	1344.464	183.4197	38.02927	1130.276	1299.649	593.238
10	7	0	0	2018-03-30		905.151	1387.213	1380.468	169.023	53.50482	1137.781	1346.475	640.862
11	5	0	0	2018-04-04		1786	1389.282	1478.042	375.8098	24.83263	773.0661	1615.843	428.156
20	12	1	0	2018-09-05		1176.147	1338.085	1297.029	274.3049	48.42406	934.9131	1269.149	444.554
1	8	1	1	2018-01-26		1235.808	1224.308	1231.955	230.2784	33.79971	944.4228	1140.633	506.756
2	9	0	1	2017-11-14		1345.825	921.0866	934.2837	64.92303	75.683	900.7168	852.0205	800.254
3	8	0	1	2017-12-12		1705.627	1308.031	1435.797	214.3683	43.26152	1104.99	1427.597	568.443
4	5	1	1	2018-01-10		1284.18	1247.689	1242.813	140.2163	27.62137	1050.129	1175.917	696.351
5	8	1	1	2018-04-02		1220.856	1057.731	980.0916	178.0119	49.42372	794.1164	784.665	524.694
6	7	1	1	2018-02-23		1542.54	1019.776	969.1109	124.1691	35.67121	832.1023	833.0869	665.247
7	12	1	1	2018-03-15		1157.51	1141.857	1055.239	178.3905	11.17743	898.5534	1032.106	932.829
12	6	1	1	2018-03-30		1056	382.863	321.2877	98.90708	62.27851	289.4459	50.57256	381.649
16	12	0	1	2018-05-08		1388.397	1062.263	1043.397	135.7453	20.45277	856.39	935.3018	700.38
17	16	1	1	2018-05-15		1213.989	1071.072	1069.117	116.7638	28.63731	930.9095	965.0491	699.683
18	11	0	1	2018-06-01		1597.137	942.431	892.9691	174.9592	52.0424	720.8341	658.829	510.449
19	5	1	1	2018-06-08		1249.585	961.6571	932.3233	41.78707	23.49551	885.7878	879.7996	866.13

measure2	area (mm2	ADC	monoexpc	f	D*	D	DDC	alpha	AST	ALT	T.bil	D.bil
	876.608	946.2982	1115.745	103.3111	4.34227	915.6463	1056.681	814.252	407	285	11.5	9
	1234.589	837.6869	912.498	111.0418	15.36209	761.4277	801.1778	744.15	195	124	8.2	6.6
	1013.489	1233.13	1320.604	166.8897	96.96406	1135.562	1255.624	596.029	441	323	5.8	4.6
	938.11	1157.936	1254.105	142.5992	11.15168	1041.567	1200.79	770.424	419	109	2.7	2.2
	442.2	838.1037	813.5029	70.68365	20.58373	763.9171	680.7147	718.5	169	82	9.2	6.9
	1242.265	999.2074	956.209	140.3006	25.15157	825.035	792.8474	605.45	137	44	9.6	7.3
	1684.605	1097.006	1164.687	186.3891	7.344	983.5519	1159.708	983.938	127	94	6.3	5.2
	985.938	1167.575	1186.088	167.8094	119.0844	1004.289	1076.28	574.601	164	126	3.4	2.9
	1672.937	1263.872	1183.837	176.4244	33.81547	925.9123	1088.796	626.58	44	21	6.4	3.2
	467.529	1422.128	1381.786	143.1448	70.14215	1203.119	1349.198	679.62	101	34	5.1	3.2
	1998.999	1422.703	1380.082	333.1565	77.23631	966.4778	1512.77	359.905	32	14	13	9.3
	618.487	243.1125	185.926	158.1995	17.8784	28.45903	243.1125	822.109	108	77	8.6	6.9
	1533.966	1143.51	1082.357	111.0737	42.96614	962.1021	986.3828	711.612	75	46	5.5	4.6
	1224.976	1065.65	1075.254	142.1818	17.96449	879.6192	970.4009	693.737	168	154	7.8	5.6
	883.026	780.079	741.9047	55.59112	52.1723	691.7004	656.1932	820.527	276	180	8.5	6.4
	1482.22	964.7911	946.2165	119.0017	21.75264	831.8156	798.437	648.004	75	41	5.7	4.7
	661.011	1332.658	1342.545	246.1306	56.47776	1032.593	1323.645	482.473	228	184	7	5.5

ALP	rGT
507	260
824	631
316	488
306	161
821	316
636	564
583	1047
1121	149
661	99
282	86
572	88
452	67
718	333
676	118
1007	471
663	403
598	188
