# Supplementary material for: Clinical utility of mono-exponential model diffusion weighted imaging using two b-values compared to the bi- or stretched exponential model for the diagnosis of biliary atresia in infant liver MRI
Source: PLoS One. 2019 Dec 18;14(12):e0226627. doi: 10.1371/journal.pone.0226627 (PMC6920030; doi:10.1371/journal.pone.0226627)
Supplement: Attachment — Submitted filename: Review_PONE-D-19-21097_ASP.docx [file pone.0226627.s002.docx]

PONE-D-19-21097: Usefulness of mono-, bi-, and stretched exponential model diffusion weighted imaging for the differentiation of biliary atresia and non-biliary atresia in pediatric liver MRI

This manuscript demonstrates feasibility and utility of multi-b-value diffusion imaging in infant population for differentiation of biliary atresia and non-biliary atresia and provides further insights into the diffuse changes in liver. One important finding of the study as written in discussion is “The ADC 2 with two b-values showed excellent diagnostic performance and was comparable with other significant parameters in this study, suggesting that image acquisition time for obtaining MEM in pediatric liver can be reduced.” Performing MRI in infants is especially challenging due to time constraints imposed by the need for sedation/anesthesia. In that light this reviewer would recommend authors to keep the primary focus of the manuscript (and Title) something like “Clinical utility of two b-values compared to mono-, bi-, and stretched exponential model diffusion weighted imaging for the differentiation of biliary atresia and non-biliary atresia in infant liver MRI”.

The systematic comparison of using 2 (0 and 800) versus 10 (0, 25, 50, 75 100, 150, 200, 400, 600, 800) b values and using mono-, bi-, and stretched exponential models to derive different measures of diffusion and pseudo-diffusion is an important endeavor. As authors have described in the discussion prognostic value of the additive quantitative information from ADC mono, f, and DDC cannot be inferred from this specific study. Additionally, in routine clinical practice added value of ADC 2 (with considerations for sedation and expensive MRI) over γGT, itself may need justification.

Paper is very well written paper. Although this is a limited sample study, the findings of the study are very promising and encouraging for further more rigorous clinical validation. Authors have done a very good job of putting their contribution in this field in right perspective in the discussion session. Overall, discussion is very well written and has good balance. However, the points from the discussion do not reflect in the conclusion. The findings do not support claim of “new imaging parameters for the diagnosis of BA”. The γGT should be mentioned in the conclusion. Same applies for the Abstract of the manuscript.

Few remarks,

Title

Title may benefit with a qualifier

Clinical utility of two b-values compared to mono-, bi-, and stretched exponential model diffusion weighted imaging for the differentiation of biliary atresia and non-biliary atresia in infant liver MRI

Abstract

The values and differentiation power of should be reported. See comments above.

Introduction

A line referring to consideration for added time for extra sequences whould add to the context of the work (ADC 2 v/s ADC mono)

Materials and Methods

L 86: Consider changing different liver MRI DWI sequences to “composition of b values”

L 129: ADC “2” refers to ADC using 2 b values, thus ADC 1 may be better referred to as ADC mono or something like that.

Discussion

Overall, discussion is very well written and has good balance.

Figures.

Please include appropriate values and units for all the color bar
